# Evaluation of Sirtuin-3 probe quality and co-expressed genes using literature cohesion

**DOI:** 10.1186/s12859-019-2621-z

**Published:** 2019-03-14

**Authors:** Sujoy Roy, Kazi I. Zaman, Robert W. Williams, Ramin Homayouni

**Affiliations:** 1 Bioinformatics Program, University of Memphis, Memphis, 38152 USA; 2 Center for Translational Informatics, University of Memphis, Memphis, 38152 USA; 3 Department of Genetics, Genomics and Informatics, University of Tennessee Health Science Center, Memphis, 38163 USA; 4 Department of Biology, University of Memphis, Memphis, 38152 USA

**Keywords:** Sirt3, Microarray, BXD mice, GeneNetwork.org, Text mining, Latent Semantic Indexing

## Abstract

**Background:**

Gene co-expression studies can provide important insights into molecular and cellular signaling pathways. The GeneNetwork database is a unique resource for co-expression analysis using data from a variety of tissues across genetically distinct inbred mice. However, extraction of biologically meaningful co-expressed gene sets is challenging due to variability in microarray platforms, probe quality, normalization methods, and confounding biological factors. In this study, we tested whether literature derived functional cohesion could be used as an objective metric in lieu of ‘ground truth’ to evaluate the quality of probes and microarray datasets.

**Results:**

We examined Sirtuin-3 (Sirt3) co-expressed gene sets extracted from either liver or brain tissues of BXD recombinant inbred mice in the GeneNetwork database. Depending on the microarray platform, there were as many as 26 probes that targeted different regions of Sirt3 primary transcript. Co-expressed gene sets (ranging from 100–1000 genes) associated with each Sirt3 probe were evaluated using the previously developed literature-derived cohesion *p*-value (LPv) and benchmarked against ‘gold standards’ derived from proteomic studies or Gene Ontology classifications. We found that the maximal F-measure was obtained at an average window size of 535 genes. Using set size of 500 genes, the Pearson correlations between LPv and F-measure as well as between LPv and mitochondrial gene enrichment *p*-values were 0.90 and 0.93, respectively. Importantly, we found that the LPv approach can distinguish high quality Sirt3 probes. Analysis of the most functionally cohesive Sirt3 co-expressed gene set revealed core metabolic pathways that were shared between hippocampus and liver as well as distinct pathways which were unique to each tissue. These results are consistent with other studies that suggest Sirt3 is a key metabolic regulator and has distinct functions in energy-producing vs. energy-demanding tissues.

**Conclusions:**

Our results provide proof-of-concept that literature cohesion analysis is useful for evaluating the quality of probes and microarray datasets, particularly when experimentally derived gold standards are unavailable. Our approach would enable researchers to rapidly identify biologically meaningful co-expressed gene sets and facilitate discovery from high throughput genomic data.

**Electronic supplementary material:**

The online version of this article (10.1186/s12859-019-2621-z) contains supplementary material, which is available to authorized users.

## Background

The amount of genome-wide gene expression data available in public repositories is accumulating rapidly. Increasing evidence suggests that genes in related metabolic pathways and cellular processes are coordinately expressed [1, 2]. Recent studies using co-expression analysis have provided important insights into complex traits and diseases and have enabled researchers to reverse engineer molecular pathways [3, 4]. The GeneNetwork database is a web resource that contains a large amount of gene expression data from a variety of tissues across panels of recombinant inbred (RI) mice, which have been derived through inbreeding of progeny from distinct inbred parental lines [5–7]. Co-expression studies using RI mice have identified gene networks associated with alcohol and stress responses [8], fear conditioning [9], liver fibrosis [10], retinal ganglion cell function [11], and many other complex traits.

Although co-expression analysis can be quite useful, there are major challenges when using microarray-based gene expression data. Microarrays are highly sensitive to biological and technical variability, which often result in noisy data [12]. In addition, different microarray platforms have variable technical reproducibility [13]. Moreover, although microarray platforms often include several probes for each target transcript, the probes produce inconsistent results due to targeting error or differences in hybridization properties [14]. It is therefore necessary to develop scalable objective methods that can identify problematic probes and datasets.

Our group has developed various literature-based semantic approaches to derive implicit functional associations between genes, transcription factors, or microRNAs [15–19]. We have demonstrated that the semantic similarity scores can be used to calculate a literature *p*-value (LPv) representing the functional cohesion of gene sets [20]. The method was shown to be both accurate and robust when evaluated against Gene Ontology (GO) classifications. Subsequently, we used LPv to compare different microarray normalization procedures [21]. More recently, an extension of this method, called Literature Based Functional Significance (LBFS), was used to evaluate statistical methods for determining differentially expressed genes from microarrays [22].

The goal of this study is to apply literature-based functional cohesion analysis to quantitatively evaluate co-expressed gene sets derived from the GeneNetwork database. In particular, we used this method to evaluate inconsistencies between probes that target the same transcript by benchmarking our method against three proteomic datasets focused on Sirtuin-3 signaling pathway. Sirtuin-3 (Sirt3) belongs to a family of NAD(+)-dependent deacetylases and plays an important role in regulation of cellular metabolism and aging [23, 24]. Sirt3 is a mitochondrial protein that is highly expressed in energy-demanding tissues such as brain, heart, skeletal muscle and kidney, as well as in energy-producing tissue such as liver. Sirt3 deficiency in mice results in a reduction of ATP production through inhibition of oxidative phosphorylation [25]. In addition, Sirt3 regulates key enzymes in fatty acid oxidation, amino acid metabolism and anti-oxidant defenses [24]. In liver, acetyl proteomic studies have demonstrated that Sirt3 is involved in global metabolic reprogramming during calorie restriction [26]. In neurons, Sirt3 is required for adaptive responses to excitotoxicity as well as oxidative and mitochondrial stress [27].

## Methods

### Gene document collection and gene-gene similarity calculation

Medline citations for 21,027 mouse genes were collected based on the PubMed identifiers (PMIDs) in the gene2pubmed repository [28] available at NCBI, and concatenated to construct a gene-document for each gene. Gene-Gene similarity scores were calculated by Latent Semantic Indexing (LSI) as previously described [15–17]. Briefly, a term-by-gene matrix was created, where the entries of the matrix were the log-entropy weighted frequencies of terms in the document collection. Then, a truncated singular value decomposition (SVD) of that matrix was performed to produce a lower dimension (reduced rank) concept-by-gene matrix with 500 concepts. Genes were then represented as concept vectors in the reduced rank matrix and the similarity between genes was calculated as the cosine of the vector angles. A graphical representation of the procedure is shown in Additional file 1: Figure S1.

### Literature Cohesion *P*-value (LPv) calculation

Literature cohesion *p*-values (LPv) for a given gene set were calculated based on LSI derived gene-gene similarities using a slightly modified version of the procedure previously described [20]. LPv is derived by using Fisher’s exact test to determine whether the number of pair-wise literature similarity associations above a pre-calculated threshold in a given gene set are significantly higher than that expected by chance. The pre-calculated threshold was set at 95^*th*^ percentile of all pairwise similarities among the 21,027 genes. For clarity, the procedure is briefly described below.

Let *S* be the set of all pairwise cosines for the set of all 21,027 genes *G*, $|S| = |G| \choose 2$. Let *T* represent 95^*th*^ percentile of all pairwise cosine similarities in *S*. For a given subset *G*^′^ of *G* for which LPv needs to be calculated, let *S*^′^ be the set of all pairwise cosines for the set *G*^′^, $|S'| = |G'| \choose 2$. Additionally, let:

sample size *k*≤50 and number of samples *n*,*N*≈1000.

$R^{\prime }_{1}, R'_{2}, \ldots R'_{n}$ be *n* randomly selected sets of cosines from *S*^′^, each the same size *k*.

*A* be the average of counts of cosines that meet the criteria |*R*1′≥*T*|,|*R*2′≥*T*|,…|*Rn*′≥*T*|.

*B* be the average of counts of cosines that meet the criteria |*R*1′<*T*|,|*R*2′<*T*|,…|*Rn*′<*T*|.

*R*_1_,*R*_2_,…*R*_*N*_ be *N* randomly selected sets of cosines from *S*, each the same size *k*.

*C* be the average of counts of cosines that meet the criteria |*R*_1_≥*T*|,|*R*_2_≥*T*|,…|*R*_*N*_≥*T*|.

*D* be the average of counts of cosines that meet the criteria |*R*_1_<*T*|,|*R*_2_<*T*|,…|*R*_*N*_<*T*|.

The literature cohesion *p*-value (LPv) is calculated using a right tailed Fisher’s exact test [29]:

1 $$ {{} \begin{aligned} p = \frac{\binom {A+B} {A} \binom {C+D} {C}}{\binom {A+B+C+D} {A+C}} = \frac{(A+B)!(C+D)!(A+C)!(B+D)!}{A!B!C!D!(A+B+C+D)!} \end{aligned}}  $$

We essentially calculate the probability of observing by random chance, cosines as high as observed in the input gene set. Lower *p*-value indicates higher cohesion. We transform the metric slightly by taking − log10(*p*-value) so that the higher value indicates higher cohesion. Additional file 1: Figure S2 demonstrates the LPv calculation procedure with a scaled down representative example.

### Workflow

**Fig. 1:**
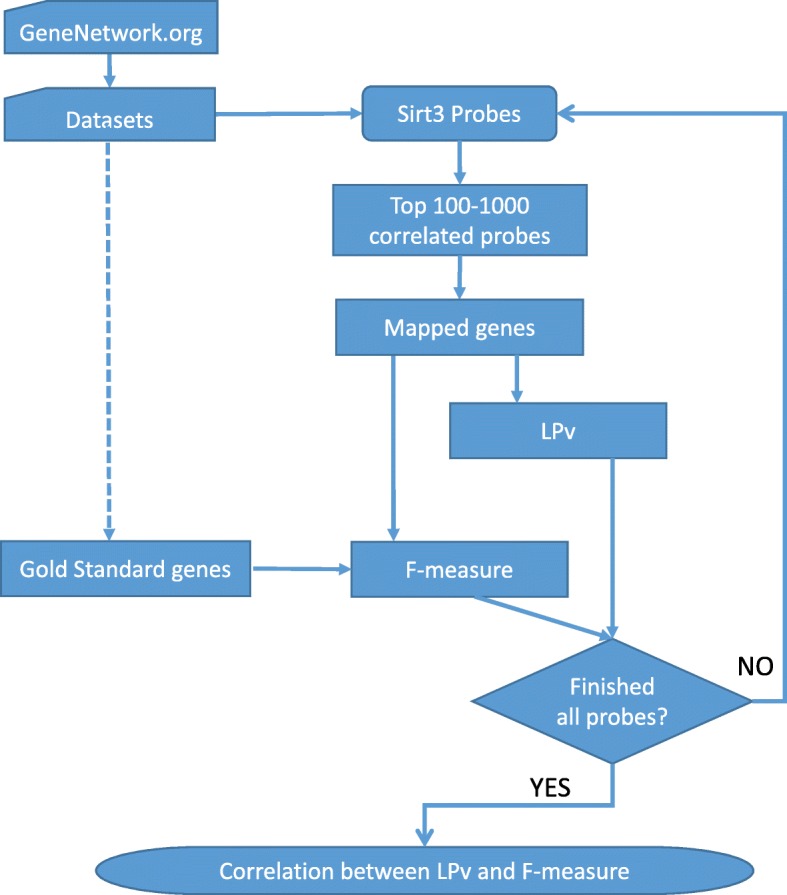
Workflow

The workflow for our approach is shown in Fig. 1. For a given tissue and dataset in GeneNetwork database, all Sirt3 probes were evaluated. For each Sirt3 probe in the dataset, 10 different Sirt3 co-expressed gene sets, containing 100 to 1000 highest correlated genes, were selected. LPv was calculated for each Sirt3 co-expressed gene set. In addition, the F-measure (F-score) of the gold standard gene sets was calculated for each Sirt3 co-expressed gene set. Lastly, the Pearson correlation coefficient was calculated for the LPvs and F-scores of all Sirt3 co-expressed gene sets.

### Microarray datasets

Three genome-wide gene expression datasets pertaining to liver (2) and brain (1) tissues across BXD recombinant inbred mice were identified from GeneNetwork. The datasets were derived from different microarray platforms and normalization methods. From each dataset, sets of 100–1000 genes whose expression patterns were maximally correlated with Sirt3 expression were extracted. The following datasets were used:

1. DS1: SUH BXD Liver CCl4-treated Affy Mouse Gene 1.0 ST (Jun11) RMA : 10568997 [10]
  Strains: 33 strains including 30 BXD strains, both parental strains (C57BL/6J, DBA/2J), and B6D2 F1 hybrids
  Platform: Affy Mouse Gene 1.0 ST (GPL6246)
  Normalization: RMA
  Probes: 10
  Tissue: Liver
2. DS2: EPFL/LISP BXD CD+HFD Liver Affy Mouse Gene 1.0 ST (Apr13) RMA Exon Level [30]
  Strains: 40 strains of the BXD family (BXD43 – BXD103) and both parental strains (C57BL/6 and DBA/2)
  Platform: Affy Mouse Gene 1.0 ST (GPL6246)
  Normalization: RMA
  Probes: 10
  Tissue: Liver
3. DS3: UMUTAffy Hippocampus Exon (Feb09) RMA Database
  Strains: 93 strains including 70 BXD inbred strains, 2 parental strains (C57BL/6J and DBA/2J), B6D2 F1, and 20 other inbred strains (129S1/SvImJ, A/J, AKR/J, BALB/cByJ, BXSB/MpJ, C3H/HeJ, CAST/EiJ, FVB/NJ, KK/HlJ, LG/J, MOLF/EiJ, NOD/LtJ, NZB/BlNJ, NZO/HlLtJ, NZW/LacJ, PWD/PhJ, and WSB/EiJ)
  Platform: Affy Mouse Exon 1.0 ST (GPL6193)
  Normalization: RMA
  Probes: 26
  Tissue: Hippocampus

## Results

### Gold standards gene sets used for benchmarking microarray datasets

**Fig. 2:**
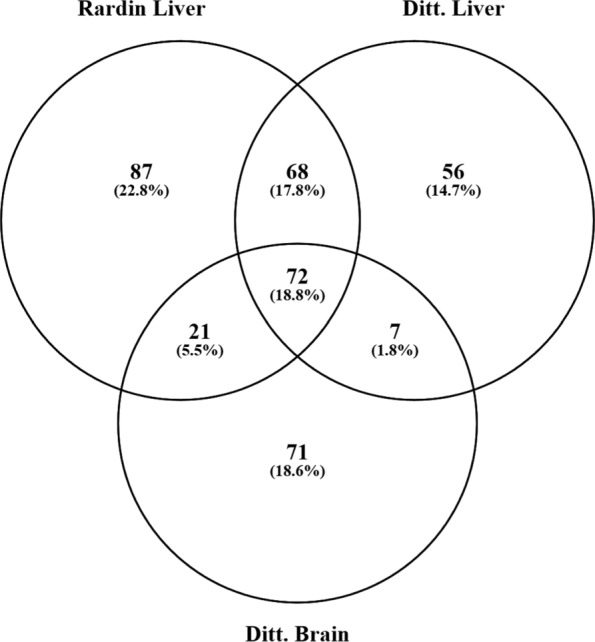
Overlap between Sirt3 targets identified in proteomic studies by Rardin et al. [31] and Dittenhafer-Reed et al. [32], henceforth referred to as Gold Standard set 1 (GS1) and 2 (GS2), respectively

Selection of an appropriate gold standard for evaluation of microarray data and gene co-expression networks is a challenging task due to a lack of ‘ground truth’. Rather than using other gene expression data, which can have overlapping confounds, we used a more functional approach for selection of gold standards. Based on the premise that co-expressed genes function together in closely linked signaling pathways, we expect that co-expressed genes directly interact with one another and are localized in the same cellular compartment. In this study, we focused on Sirtuin-3 (Sirt3) which is a mitochondrial NAD-dependent deacetylase that broadly controls cellular metabolism and has been implicated in a variety of diseases and aging-related processes [23, 24]. For benchmarking purposes, we used two different proteomic studies, which identified direct targets of Sirt3 by comparing the acetylomes in different tissues of Sirt3 knock-out and wild-type control mice. Rardin et al. [31] identified 248 proteins whose acetylation levels were significantly (*p*<0.05) increased in Sirt3 knock-out livers (Gold Standard 1, GS1). Using a similar approach, Dittenhafer-Reed et al. [32] identified 203 Sirt3 targets (Golds Standard 2, GS2) in liver. Between the two studies, 140 Sirt3 targets were common in liver tissue (Fig. 2). To benchmark Sirt3 co-expression networks in the hippocampus, only one acetyl proteomic dataset could be found. Dittenhafer-Reed et al. [32] identified 171 proteins whose acetylation levels were significantly changed in Sirt3 KO brains. Interestingly, 93 and 79 out of the 171 Sirt3 target proteins in brain were also found in liver according to Rardin et al. and Dittenhafer-Reed et al., respectively (Fig. 2). As an alternative functional benchmarking approach, we examined mitochondrial enrichment of various Sirt3 co-expressed gene sets.

### Literature cohesion of Sirt3 co-expressed gene sets in liver

We initially focused on two liver datasets in GeneNetwork that used Affymetrix Gene 1.0 ST arrays, which contained 10 different Sirt3 probes. While each of the probes appropriately targeted Sirt3 exons, we found that the correlation values among the 10 Sirt3 probes across the panel of BXD RI mice were highly variable (Additional file 1: Figure S3). Only 6 Sirt3 probes showed a Pearson correlation >0.5 for GS1, and 4 probes showed a Pearson correlation >0.5 for GS2. These results suggest that using probe-to-probe correlations may not necessarily be a good metric to evaluate probe quality as a whole, since the same probes showed different correlation structure in different datasets (Additional file 1: Figures S3A and S3B).

We next examined the correlated gene sets produced by the different Sirt3 probes. We found that the co-expressed gene sets associated with highly inter-correlated Sirt3 probes (e.g., PID1056899) were dramatically different from the Sirt3 probes which were uncorrelated (PID 10569006; Additional file 1: Figure S4). Whereas the strongly inter-correlated Sirt3 probes identified robust gene sets with high connectivity (Pearson correlation >0.8 or <−0.8) among themselves (Additional file 1: Figure S4A),

the weakly correlated Sirt3 probes produced gene sets that were weakly connected (Additional file 1: Figure S4B).

**Fig. 3:**
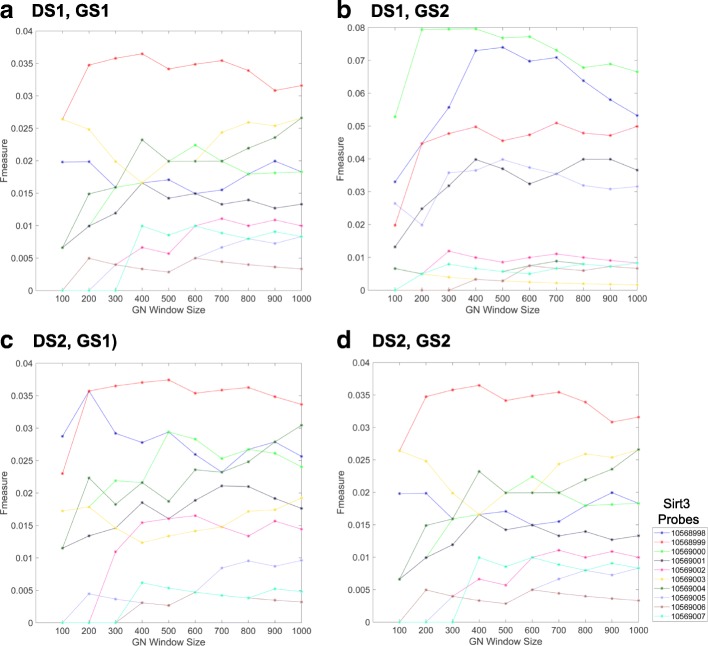
Relationship between gold standard F-measure and gene co-expression window size. The F-scores were calculated for the top 100–1000 co-expressed genes obtained from each Sirt3 probe in two different liver datasets (DS): 1) SUH BXD CCL4 Affymetrix Gene 1.0 ST treated (**a, b**) and 2) EFPL/LISP BXD CD+HFD Affymetrix Gene 1.0 ST (**c, d**). F-measure values were calculated using two different Sirt3 gold standard gene sets: GS1, [31], (**a, c**) and GS2, [32], (**b, d**)

To evaluate which Sirt3 probes produced biologically meaningful co-expressed gene sets, we benchmarked the top 100 to 1000 correlated genes associated with each Sirt3 probe using the two proteomic Gold Standard gene sets described above. As expected, the recall of gold standard genes increased with increasing window sizes (larger co-expressed gene sets), whereas the precision decreased (Additional file 1: Figures S5 and S6). Using F-measure (weighted harmonic mean of precision and recall), we found that some probes consistently performed better across all window sizes (Fig. 3).

**Fig. 4:**
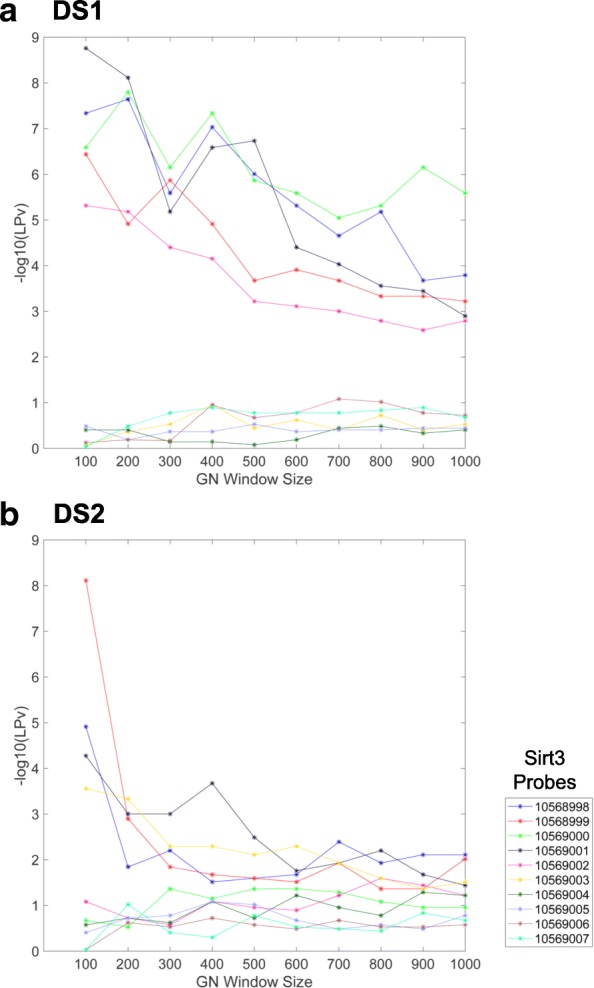
Literature derived cohesion *p*-values (LPv) for various gene co-expression window sizes. LPv was calculated for gene set sizes ranging from 100–1000 for each Sirt3 probe on the Affymetrix Gene 1.0 ST array for two different liver datasets: DS1, SUH BXD CCL4 Affymetrix Gene 1.0 ST treated (**a**) and DS2, EFPL/LISP BXD CD+HFD Affymetrix Gene 1.0 ST (**b**)

To evaluate if the Sirt3 correlated gene sets represent functionally related genes, we compared the literature derived cohesion *p*-values (LPvs) as described previously by our group [20]. LPv was calculated for gene sets ranging from 100 to 1000 genes that were associated with each Sirt3 probe in the two datasets. In general, we found that the most highly correlated genes (smaller window size) to Sirt3 probes were more functionally cohesive and that − log10(*LPv*) decreased (less significant) with increasing window size. Interesting, in DS1, only five out of the 10 Sirt3 probes produced significant (*LPv*<0.05) functional cohesion (Fig. 4). The Sirt3 co-expressed gene sets produced from four of these five probes exhibited the highest F-scores for the two gold standard gene sets (GS1 and DS2).

**Fig. 5:**
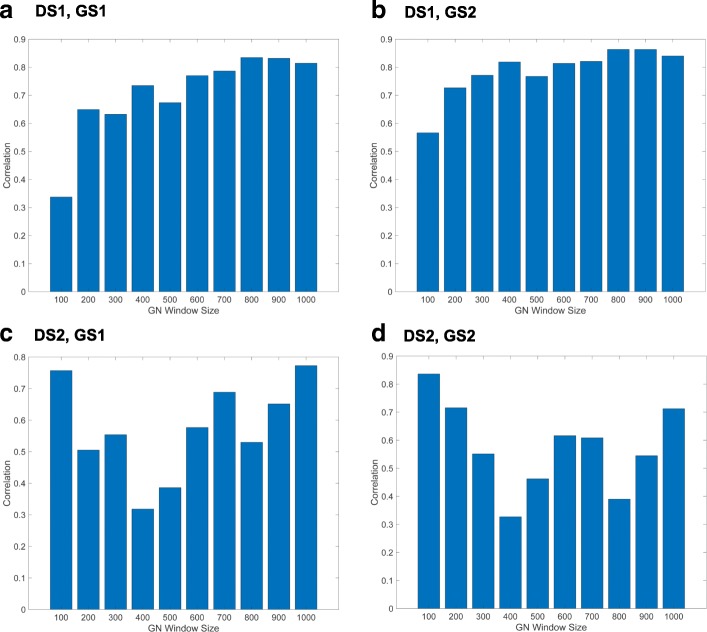
Correlation between literature derived cohesion *p*-values (LPv) and F-measure across various gene co-expression set sizes. Correlations were calculated using two gold standard sets (GS1 and GS2) and two different liver datasets (DS1 and DS2). DS1, SUH BXD CCL4 Affymetrix Gene 1.0 ST treated; DS2, EFPL/LISP BXD CD+HFD Affymetrix Gene 1.0 ST; GS1, [31]; GS2, [32]

Next, we examined the correlation between F-measure and LPv across various window sizes of Sirt3 co-expressed gene sets. Whereas the correlation between F-measure and LPv improved with increasing window size for DS1, the correlation decreased for DS2 (Fig. 5).

### Literature cohesion of Sirt3 co-expressed gene sets in brain hippocampus

**Fig. 6:**
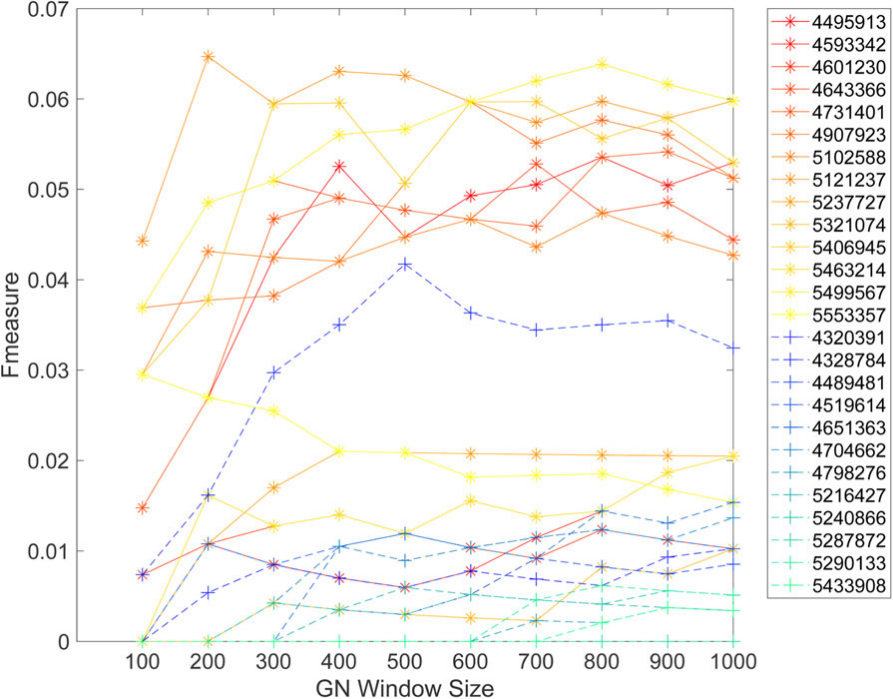
Relationship between F-measure and gene co-expression set sizes ranging from 100 to 1000 for 14 exonic (yellow/orange) and 12 intronic (blue/green) Sirt3 probes on Affymetrix Murine Exon Array

**Fig. 7:**
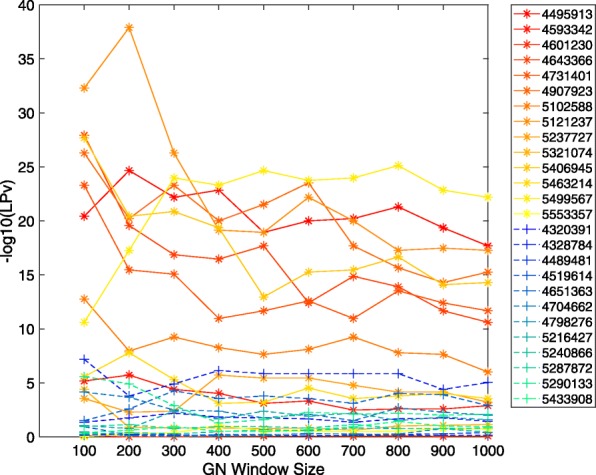
Literature derived cohesion *p*-values (LPv) for gene co-expression set sizes ranging from 100 to 1000 for 14 exonic (yellow/orange) and 12 intronic (blue/green) Sirt3 probes on Affymetrix Murine Exon Array

**Fig. 8:**
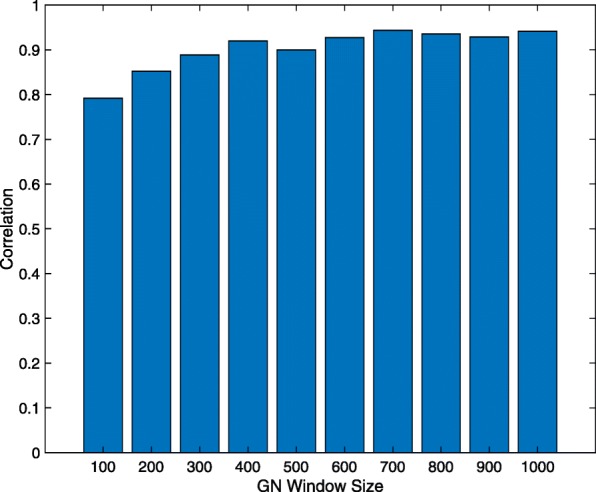
Correlation between literature derived cohesion *p*-values (LPv) and F-measure across various Sirt3 co-expression set sizes in the hippocampus

**Fig. 9:**
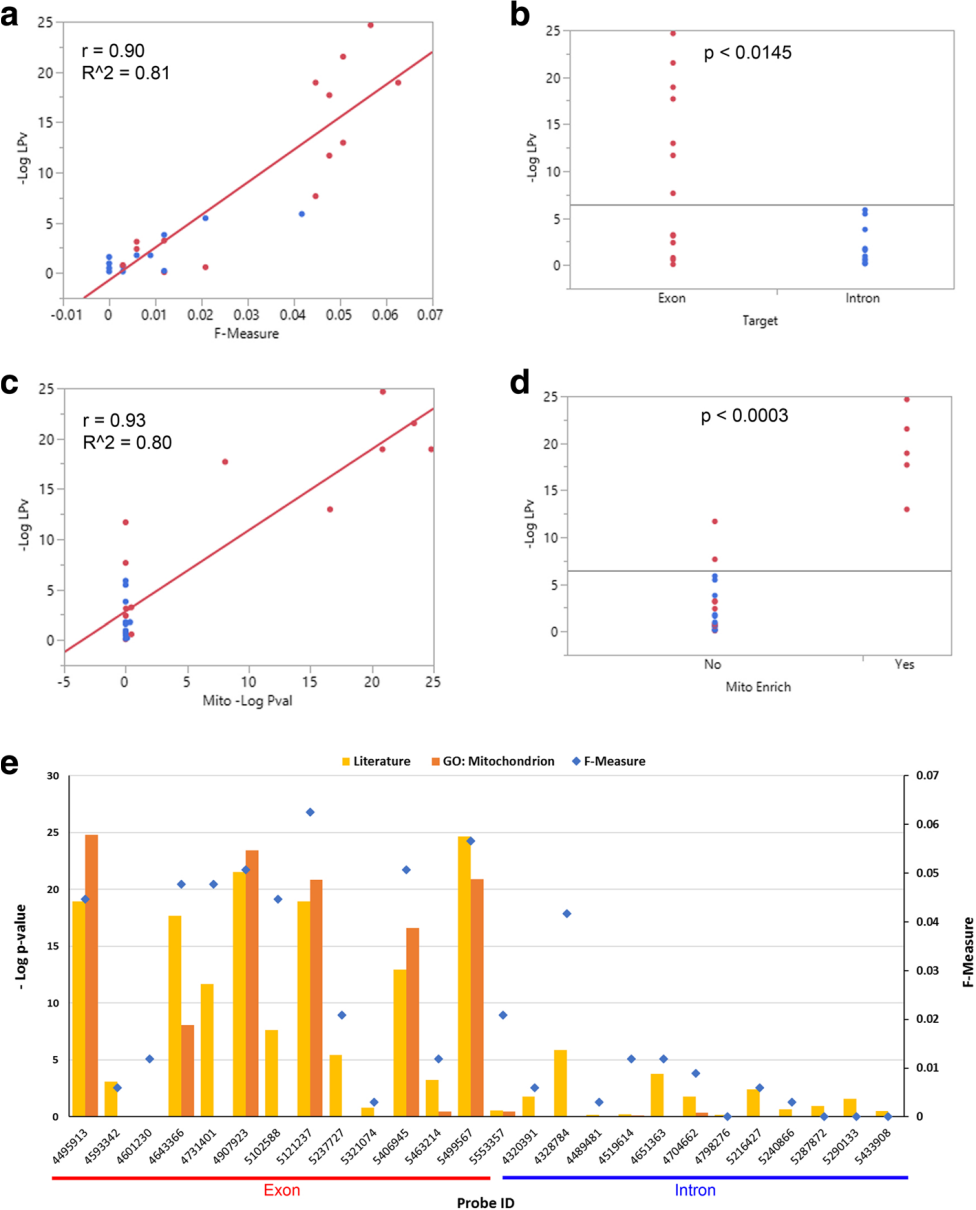
**a** Relationship between literature cohesion *p*-value (LPv) and gold standard F-measure using the top 500 Sirt3 correlated genes obtained from 14 exonic (red circles) and 12 intronic (blue circles) Sirt3 probes. **b** Distribution of LPv for Sirt3 exonic (red circles) and intronic (blue circles) probes, *p*-values were determined using Wilcoxon/Kruskal-Wallis Rank Sum test. **c** Relationship between LPv and GO:Mitochondrion enrichment *p*-value using the top 500 Sirt3 correlated genes obtained from 14 exonic (red circles) and 12 intronic (blue circles) Sirt3 probes. **d** Distribution of LPv for Sirt3 probe co-expressed gene sets which were or were not enriched for GO:Mitochondrion category, *p*-values were determined using Wilcoxon/Kruskal-Wallis Rank Sum test. **e** Bar plot showing the LPv, F-Measure and Mitochondrial enrichment for each Sirt3 probe

Multi-tissue proteomic experiments performed by [32] suggested that although Sirt3 targets a set of core mitochondrial proteins, it targets different proteins in energy-producing tissues compared to energy-demanding tissues. Therefore, we compared Sirt3 co-expressed gene sets derived from brain hippocampus in addition to liver tissues. The hippocampal study (DS3) utilized Affymetrix Exon array platform to measure transcript levels across a panel of 93 inbred mouse strains. There is a total of 26 Sirt3 probes represented in the mouse Affymetrix Exon Array; 12 probes that target intronic regions and 14 probes that target exonic regions of Sirt3 as annotated by the manufacturer. This is useful for evaluation, enabling us to use the intronic probe correlated gene sets as negative controls. For this analysis, only one proteomic gold standard dataset specific to brain tissue could be obtained [32]. In general, the F-scores of the Sirt3 exonic probe correlated gene sets were much higher than those obtained from the intronic probes (Fig. 6). This result was consistent with the LPv for the exonic probe correlated gene sets, although a few exonic probes produced insignificant literature cohesion and several intronic probes produced significant literature cohesion (Fig. 7). The highest F-scores were obtained with an average window size of 535 genes across all Sirt3 exonic probes. Focusing on a window size of 500 transcripts, we found that the majority (78%) of Sirt3 co-expressed gene sets obtained from exonic probes were significantly (*LPv*<0.05) cohesive (Table 1). Surprisingly, 6 out of 12 intronic Sirt3 probes produced significant literature cohesion although their F-scores were very low 0–0.042 (Table 1). Overall, the Pearson correlation between gold standard F-measure and − log10(*LPv*) was greater than 0.79 for all Sirt3 correlated gene set sizes ranging from 100 to 1000 (Fig. 8). For example, the Pearson correlation between F-score and − log10(*LPv*) for 500 Sirt3 co-expressed genes was 0.90 (*R*^2^=0.81). Examination of the scatter plot of the data showed that only the Sirt3 exonic probe correlated gene sets produced the highest F-scores and literature cohesion significance (Fig. 9a). Indeed the LPv of Sirt3 exonic probe correlated genes were significantly (*p*<0.0145, Wilcoxon/Kruskal-Wallis Rank Sum test) higher than the LPv of Sirt3 intronic probes (Fig. 9b).

**Table 1:** LPv and F-measure values for the top 500 Sirt3 correlated genes using different Sirt3 probes and datasets

Dataset	Probe ID	Probe target	GS1 F-measure	GS2 F-measure	LPv
*DS1: SUH BXD Liver CCl4-treated Affy Mouse Gene 1.0 ST (Jun11) RMA : 10568997*					
	**10569001**	**Exon**	**0.027**	**0.037**	**1.85E-07**
	**10568998**	**Exon**	**0.067**	**0.074**	**9.81E-07**
	**10569000**	**Exon**	**0.078**	**0.077**	**1.36E-06**
	**10568999**	**Exon**	**0.048**	**0.046**	**2.12E-04**
	**10569002**	**Exon**	**0.008**	**0.009**	**6.02E-04**
	10569007	Exon	0.003	0.006	1.66E-01
	10569006	Exon	0.011	0.003	2.12E-01
	10569005	Exon	0.045	0.040	2.95E-01
	10569003	Exon	0.005	0.003	3.59E-01
	10569004	Exon	0.003	0.006	8.30E-01
*DS2: EPFL/LISP BXD CD+HFD Liver Affy Mouse Gene 1.0 ST (Apr13) RMA Exon Level*					
	**10569001**	**Exon**	**0.016**	**0.014**	**3.24E-03**
	**10569003**	**Exon**	**0.013**	**0.020**	**7.77E-03**
	**10568998**	**Exon**	**0.029**	**0.017**	**2.54E-02**
	**10568999**	**Exon**	**0.037**	**0.034**	**2.54E-02**
	**10569000**	**Exon**	**0.029**	**0.020**	**4.33E-02**
	10569005	Exon	0.003	0.003	9.58E-02
	10569002	Exon	0.016	0.006	1.11E-01
	10569007	Exon	0.005	0.009	1.66E-01
	10569004	Exon	0.019	0.020	1.88E-01
	10569006	Exon	0.003	0.003	2.66E-01
*DS3: UMUTAffy Hippocampus Exon (Feb09) RMA Database*					
	**5499567**	**Exon**	**-**	**0.057**	**2.17E-25**
	**4907923**	**Exon**	**-**	**0.051**	**3.00E-22**
	**4495913**	**Exon**	**-**	**0.045**	**1.15E-19**
	**5121237**	**Exon**	**-**	**0.063**	**1.15E-19**
	**4643366**	**Exon**	**-**	**0.048**	**2.05E-18**
	**5406945**	**Exon**	**-**	**0.051**	**1.09E-13**
	**4731401**	**Exon**	**-**	**0.048**	**2.09E-12**
	**5102588**	**Exon**	**-**	**0.045**	**2.27E-08**
	**4328784**	**Intron**	**-**	**0.042**	**1.36E-06**
	**5237727**	**Exon**	**-**	**0.021**	**3.54E-06**
	**4651363**	**Intron**	**-**	**0.012**	**1.62E-04**
	**5463214**	**Exon**	**-**	**0.012**	**6.02E-04**
	**4593342**	**Exon**	**-**	**0.006**	**7.74E-04**
	**5216427**	**Intron**	**-**	**0.006**	**4.06E-03**
	**4320391**	**Intron**	**-**	**0.006**	**1.74E-02**
	**4704662**	**Intron**	**-**	**0.009**	**1.74E-02**
	**5290133**	**Intron**	**-**	**0.000**	**2.54E-02**
	5287872	Intron	-	0.000	1.11E-01
	5321074	Exon	-	0.003	1.66E-01
	5240866	Intron	-	0.003	2.12E-01
	5553357	Exon	-	0.021	2.66E-01
	5433908	Intron	-	0.000	3.26E-01
	4519614	Intron	-	0.012	6.10E-01
	4489481	Intron	-	0.003	7.13E-01
	4798276	Intron	-	0.000	7.13E-01
	4601230	Exon	-	0.012	8.30E-01

Probes that produced co-expressed gene sets with significant (*p*<0.05) literature cohesion are in bold font

As an alternate functional benchmarking approach, we examined the correlation between LPv and enrichment *p*-value of mitochondrial genes annotated in GO database. Consistent with the proteomic benchmarking results, we found a very high correlation of 0.93 (*R*^2^=0.80) between LPv and mitochondrial gene enrichment for Sirt3 co-expressed genes (Fig. 9c). Notably, not all Sirt3 exonic probe correlated genes and none of the intronic probe correlated genes were enriched for the Mitochondrion GO category (Fig. 9c). The LPv for the mitochondrial enriched Sirt3 correlated genes was significantly (*p*<0.0003, Wilcoxon/Kruskal-Wallis Rank Sum test) higher than the LPv for Sirt3 correlated genes which were not enriched for mitochondrial genes (Fig. 9d). Moreover, the correlation between LPv and all mitochondrial GO categories for Sirt3 correlated gene sets was significantly (*p*<10^−25^, Wilcoxon/Kruskal-Wallis Rank Sum test) higher than the correlation of LPv and all other GO categories (Additional file 1: Figure S7).

### Functional analysis of Sirt3 co-expressed gene sets in brain and liver

Having identified the most functionally cohesive Sirt3 co-expressed gene sets in liver (using PID# 10569000 in Affymetrix Mouse Gene 1.0 ST array) and hippocampus (using PID# 5499567 in Affymetrix Mouse Exon array) tissues, we performed functional enrichment analysis on the top 500 Sirt3 correlated transcripts. The most enriched categories were the UniProt Keywords *Acetylation* and *Mitochondrion cellular compartment* (Table 2). The Sirt3 co-expressed gene sets in both liver and Hippocampus were enriched for core metabolic processes such as *oxidation-reduction process* and *oxidative phosphorylation*. Interestingly, Sirt3 co-expressed genes in both tissues were associated with *focal adhesion*, *myelin sheath*, *Huntington’s disease*, *Parkinson’s disease* and *Alzheimer’s disease*. However, in the liver, the Sirt3 co-expressed genes were involved in *tricarboxylic acid cycle* and *nucleotide binding*, as well as *amino acid*, *lipid*, and *cholesterol metabolism*. In contrast, the Sirt3 co-expressed gene sets in the hippocampus were enriched for *ribosomal proteins*, *translation regulation*, *proteasome regulation* as well as *non-alcoholic fatty liver disease* and *vesicle-mediated transport pathways*.

**Table 2:** Enriched functional categories for the top 500 Sirt3 correlated genes obtained from two different datasets

Dataset	Database_category	Term	Gene count	% of input genes	*p*-value	Adj *p*-value
*DS1: SUH BXD Liver CCl4-treated Affy Mouse Gene 1.0 ST (Jun11) RMA : 10568997 (263 Gene IDs)*						
	UP_KEYWORDS	Acetylation	102	0.39	8.29E-24	2.11E-21
	GOTERM_CC_DIRECT	GO:0005739 mitochondrion	76	0.29	1.47E-21	4.79E-19
	GOTERM_BP_DIRECT	GO:0055114 oxidation-reduction process	31	0.12	1.68E-08	2.50E-05
	**KEGG_PATHWAY**	**mmu00280:Valine leucine and isoleucine degradation**	**10**	**0.04**	**3.80E+00**	**3.81E-04**
	**GOTERM_MF_DIRECT**	**GO:0000166 nucleotide binding**	**49**	**0.19**	**1.80E-05**	**5.13E-03**
	KEGG_PATHWAY	mmu05016:Huntington’s disease	16	0.06	1.84E-05	1.81E-03
	**GOTERM_BP_DIRECT**	**GO:0006629 lipid metabolic process**	**20**	**0.08**	**2.14E-05**	**6.37E-03**
	KEGG_PATHWAY	mmu00190:Oxidative phosphorylation	13	0.05	3.65E-05	1.79E-03
	**GOTERM_BP_DIRECT**	**GO:0006099 tricarboxylic acid cycle**	**6**	**0.02**	**4.31E-05**	**9.15E-03**
	GOTERM_CC_DIRECT	GO:0043209 myelin sheath	12	0.05	4.69E-05	1.69E-03
	GOTERM_CC_DIRECT	GO:0005925 focal adhesion	17	0.06	6.08E-05	1.80E-03
	KEGG_PATHWAY	mmu05012:Parkinson’s disease	13	0.05	7.23E-05	2.83E-03
	**GOTERM_BP_DIRECT**	**GO:0042632 cholesterol homeostasis**	**7**	**0.03**	**2.26E-04**	**3.68E-02**
	KEGG_PATHWAY	mmu05010:Alzheimer’s disease	13	0.05	3.68E-04	1.20E-02
*DS3: UMUTAffy Hippocampus Exon (Feb09) RMA Database (307 Gene IDs)*						
	UP_KEYWORDS	Acetylation	156	0.51	1.33E-50	3.21E-48
	GOTERM_CC_DIRECT	GO:0005739 mitochondrion	85	0.28	3.49E-22	1.19E-19
	**GOTERM_MF_DIRECT**	**GO:0003735 structural constituent of ribosome**	**30**	**0.10**	**4.17E-19**	**1.80E-16**
	**GOTERM_BP_DIRECT**	**GO:0006412 translation**	**36**	**0.12**	**1.76E-18**	**1.72E-15**
	KEGG_PATHWAY	mmu05016:Huntington’s disease	26	0.08	1.17E-12	6.36E-11
	KEGG_PATHWAY	mmu05012:Parkinson’s disease	20	0.07	8.29E-11	3.01E-09
	KEGG_PATHWAY	mmu00190:Oxidative phosphorylation	19	0.06	3.81E-10	1.04E-08
	**KEGG_PATHWAY**	**mmu03050:Proteasome**	**13**	**0.04**	**4.95E-10**	**1.08E-08**
	KEGG_PATHWAY	mmu05010:Alzheimer’s disease	20	0.07	7.75E-09	1.41E-07
	**KEGG_PATHWAY**	**mmu04932:Non-alcoholic fatty liver disease (NAFLD)**	**18**	**0.06**	**5.30E-08**	**8.25E-07**
	GOTERM_BP_DIRECT	GO:0055114 oxidation-reduction process	29	0.09	6.55E-06	1.29E-03
	**GOTERM_BP_DIRECT**	**GO:0016192 vesicle-mediated transport**	**14**	**0.05**	**6.26E-05**	**1.02E-02**
	GOTERM_CC_DIRECT	GO:0043209 myelin sheath	13	0.04	6.97E-05	1.48E-03
	GOTERM_CC_DIRECT	GO:0005925 focal adhesion	16	0.05	0.0017766	2.71E-02

The gene count, % of input gene list, raw *p*-value, and Benjamini-Hochberg adjusted *p*-value are shown for each functional category. Categories that are unique to one dataset are bolded

## Discussion

In this study, we demonstrated that using a literature-based method to determine functional cohesion of gene sets is an effective approach to evaluate the quality of specific microarray probes and their correlated gene expression. Using two different proteomic gold standard gene sets, we found that the literature derived *p*-values of Sirt3 co-expressed genes were highly correlated with F-scores across different window sizes (Figs. 5, 8 and 9). In addition, the LPv of Sirt3 co-expressed genes was highly correlated with enrichment of mitochondrial genes (Fig. 9). These results suggest that LPv may be used as a proxy benchmarking tool when gold standards are not available.

We found that not all probes targeting the same gene on a microarray platform produced meaningful co-expressed gene sets based on LPv, F-measure, or GO enrichment analysis (Figs. 3, 4, 6, 7, and 9). These results suggest that a considerable number of microarray probes may produce erroneous results. Our results are consistent with various other studies which have documented non-specific probe hybridization and other factors that affect the quality of expression analysis using Affymetrix GeneChip arrays [12, 14, 33]. Importantly, based on probe level analysis of the Hippocampus Affymetrix Exon Arrays, we found three Sirt3 exonic probes with non-significant LPv (Fig. 9, Table 1). Further analysis revealed that two of these probes (PID# 5553357 and PID# 5321074) actually target Sirt3 introns rather than exons, revealing annotation errors by the manufacturer. In addition, the third Sirt3 probe (PID# 4601230) targeted other regions in the genome in addition to Sirt3. In contrast, some Sirt3 intronic probe correlated gene sets showed significant LPv (Table 1). While the reasons for this result is unclear, it does indicate that a metric other than LPv cutoff of *p*<0.05 is needed to identify high quality probes. Based on the distribution of LPv for exonic and intronic Sirt3 probes, we found that the mean − log10(*LPv*) (6.42) may be adequate to distinguish high quality probes from intronic controls (Fig. 9b). Applying this metric to DS1 and DS2, which used Affymetrix Gene 1.0 ST array, identified five Sirt3 probes with greater than average LPv (Additional file 1: Figure S8). Four out of the five ‘high’ quality Sirt3 probes were consistent between the two datasets, suggesting that some differences in literature cohesion may be caused by experimental differences. Further study using a larger number of datasets for a given array platform will be needed to develop a global probe quality metric based on LPv.

In general, the F-scores across all probes and datasets were low (<0.04) although some co-expressed gene sets exhibited highly significant literature cohesion. The low F-measure is a result of both low recall and precision of the gold standard genes, which is likely due to a number of factors. First, low F-scores may simply be due to technical or experimental variation in the proteomic studies that we used to define the gold standards. Only 140 (56% of GS1 and 69% of GS2) Sirt3 target protein/genes in the liver were common between the two proteomic studies (Fig. 2). Second, low F-scores may be explained by the fact that not all Sirt3 co-expressed genes are expected to be direct deacetylation targets of Sirt3, yet they may play important roles in Sirt3 signaling pathways. Third, gene expression is not expected to tightly correlate with protein levels nor post-translational modification. Fourth, the liver samples used for our co-expression analysis were from treated animals, whereas the gold standard genes were identified from untreated Sirt3 knock-out and wild-type animals. Therefore, it is possible that many of the Sirt3 co-expressed genes in the liver datasets may be confounded by the treatment. Finally, since the gold standard experiments utilized Sirt3 KO animals, some of the acetylation changes may be a result of indirect affects or compensatory mechanisms caused by Sirt3 deficiency during development. Nevertheless, using two different sets of proteomic gold standards as well as benchmarking with mitochondrial enrichment analysis enabled us to validate LPv performance across multiple tissues and datasets. As noted above, we consistently found high correlation between LPv and the different benchmarks (Figs. 5, 8 and 9).

Large-scale acetyl proteomic studies have reported that Sirt3 regulates a wide range of metabolic enzymes and is responsible for global metabolic reprogramming [26, 31, 32]. Consistent with these studies, our functional enrichment analysis of Sirt3 co-expressed genes in BXD recombinant inbred mice revealed that Sirt3 participate in core metabolic pathways involved in oxidative phosphorylation, however the specific targets in this biochemical pathway were different in the hippocampus compared to liver. For example, out of 31 liver genes and 19 brain genes involved in oxidative phosphorylation pathway, only 3 were common between liver and hippocampus. This suggest that although Sirt3 generally regulates oxidative phosphorylation, it may mediate its effects through distinct target proteins in different tissues. Also, consistent with previous proteomic studies, our analysis showed that Sirt3 regulates distinct metabolic pathways in an energy producing tissue such as liver compared to an energy demanding tissue such as brain hippocampus. In the liver, we found that Sirt3 co-expressed genes are involved in fatty acid and amino acid metabolism, whereas in the brain Sirt3 co-expressed genes are involved in regulation of protein synthesis and vesicle mediated transport. This result is consistent with recent studies that reported Sirt3 is critical for adaptive responses to exercise and metabolic challenges in neurons [27].

Using a literature-based approach such as LPv is important because gold standard sets (despite their limitations discussed above) are not readily available for the vast majority of genes. In the absence of gold standards, it is difficult to assess the quality of different probes for a given gene represented on various microarray platforms. Lack of appropriate quality benchmarking will lead to false discovery and hinder biological interpretation of the data. Thus, we posit that the LPv approach would enable researchers to focus on the best probes and datasets and ultimately facilitate genomic discovery. The GeneSet Cohesion Analysis Tool (GCAT) [20] which calculates literature derived functional cohesion *p*-values is readily available in GeneNetwork.org.

## Conclusions

Taken together, we have demonstrated that literature derived functional cohesion provides for a robust, automated and objective metric for evaluating the quality of probes and co-expressed genes. This makes the LPv metric a viable probe quality indicator substitute in the absence of ‘ground truth’ or experimentally derived gold standards, which is the case for most genes.

## Additional file

Additional file 1 ’Roy_et_al_Additional_file_1.pdf’ contains **Figures S1-S8**. (PDF 8187 kb)
