# Supplementary material for: Evaluation of Sirtuin-3 probe quality and co-expressed genes using literature cohesion
Source: BMC Bioinformatics. 2019 Mar 14;20(Suppl 2):104. doi: 10.1186/s12859-019-2621-z (PMC6419539; doi:10.1186/s12859-019-2621-z)
Supplement: Supplementary file 1 — ’Roy_et_al_Additional_file_1.pdf’ contains Figures S1-S8. (PDF 8187 kb) [file 12859_2019_2621_MOESM1_ESM.pdf]

Supplementary Material: Evaluation of Sirtuin-3 Co-expression Networks using Literature Cohesion

1 SUPPLEMENTARY FIGURES

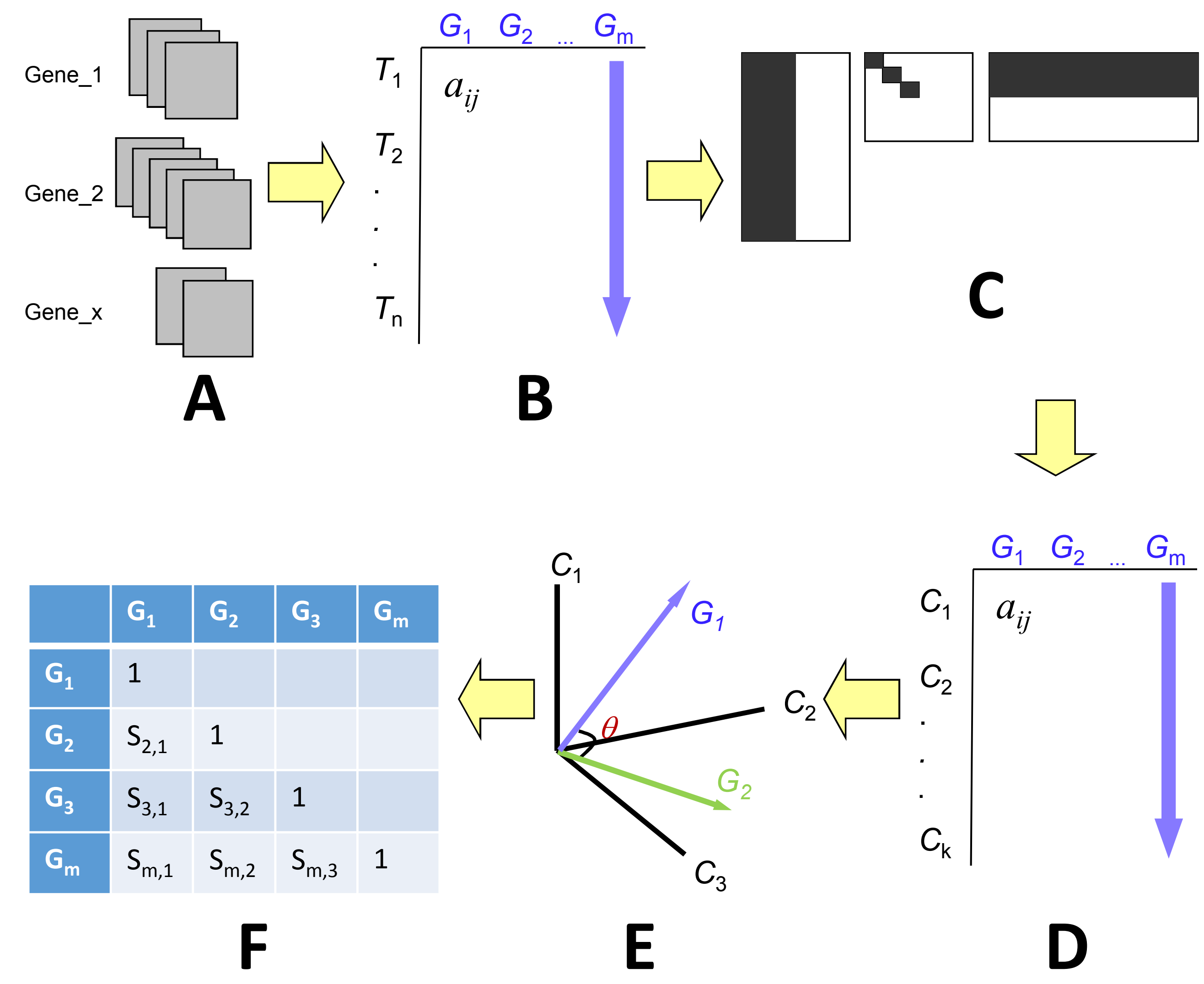

Figure S1. Overview of the LSI based procedure to calculate implicit gene similarity values between genes. (**A**) Gene documents were created for each of the 21027 genes in the mouse genome by concatenating titles and abstracts corresponding to the genes. (**B**) The documents were parsed and a term-by-gene matrix was created, the entries of which contained term frequencies in the gene documents. (**C**) The matrix was normalized and decomposed into two component matrices using SVD. (**D**) One of the component matrices represented genes as vectors of concepts. (**E**) The similarity between any two genes was calculated as the cosine between their gene vectors. (**F**) Cosines were calculated for all possible gene pairs, which were stored in a cosine similarity association matrix.

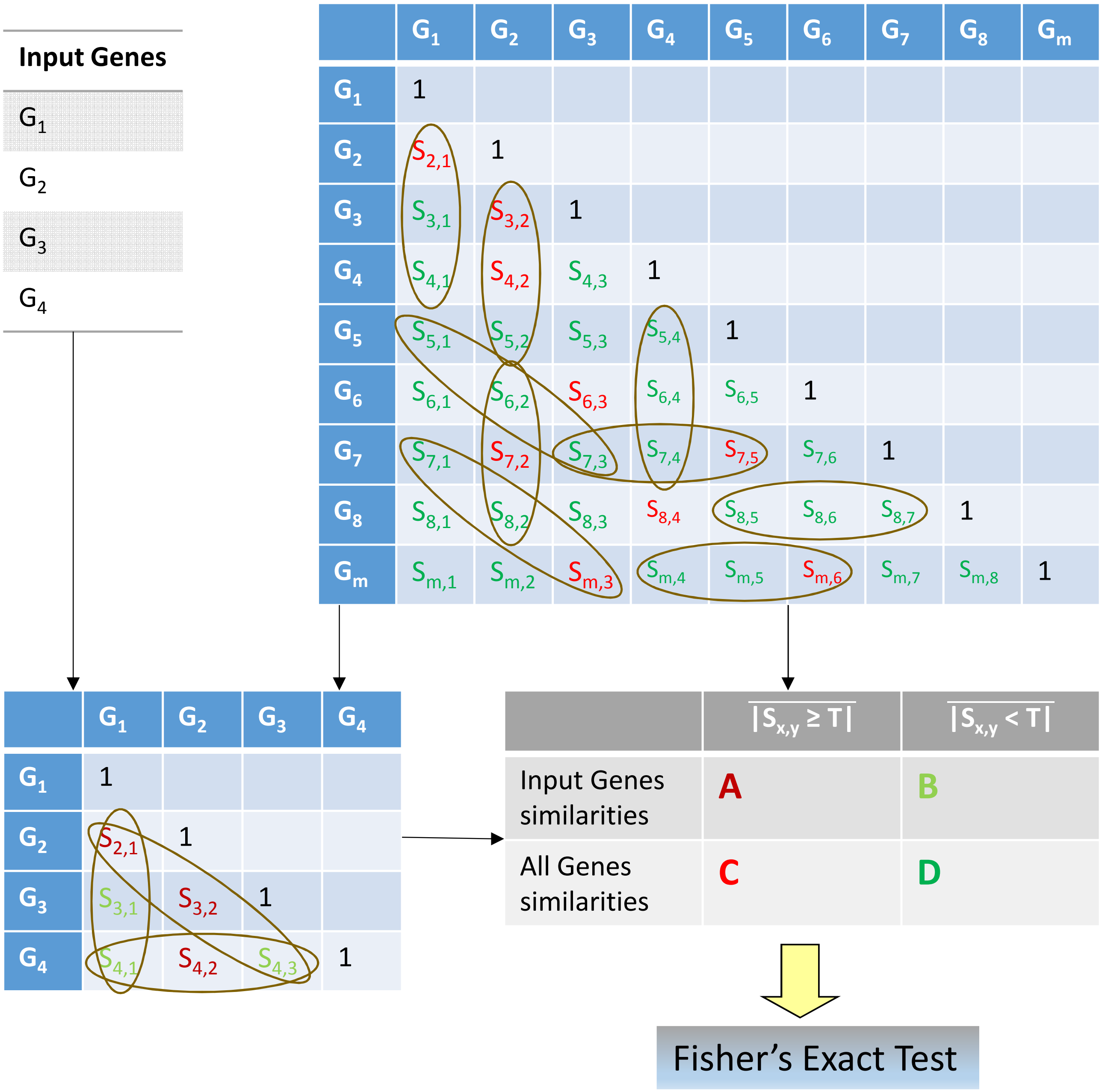

Figure S2. Overview of LPv calculation. The figure demonstrates the literature cohesion calculation procedure for random sample size = 3. For a given input subset of genes, the corresponding cosine similarity submatrix was extracted from the larger cosine similarity matrix for all genes under consideration. From the submatrix, random samples of similarities were extracted. For each sample in the submatrix, counts of similarity values greater than or equal to a similarity threshold (T) were calculated and averaged across samples (depicted as A). Counts of similarity values less than the threshold were calculated as well and averaged across samples (depicted as B). The process was repeated for the large matrix to get C and D averaged counts. A Fisher's exact text was calculated to generate the literature cohesion p-value.

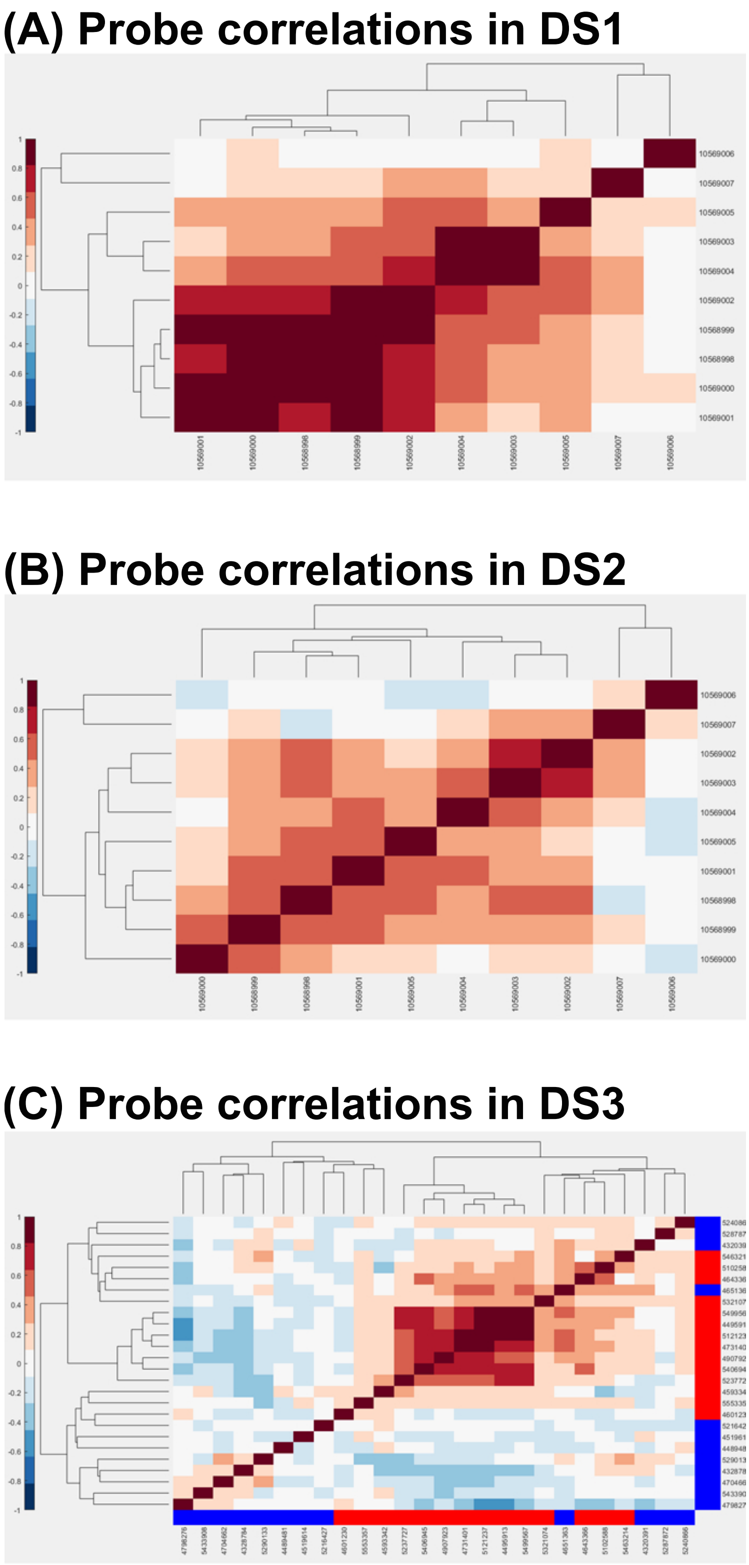

Figure S3. Sirt3 Probe correlations. (**A**) Correlations among 10 exonic Sirt3 probes in liver samples in SUH dataset (DS1). (**B**) Correlations among 10 exonic Sirt3 probes in liver samples in EFPL/LISP dataset (DS2). (**C**) Correlations among 26 exonic and intronic Sirt3 probes in hippocampus dataset (DS3). Red categories denote exonic probes and blue categories denote intronic probes.

(A) Sirt3 PID10568999 co-expression network

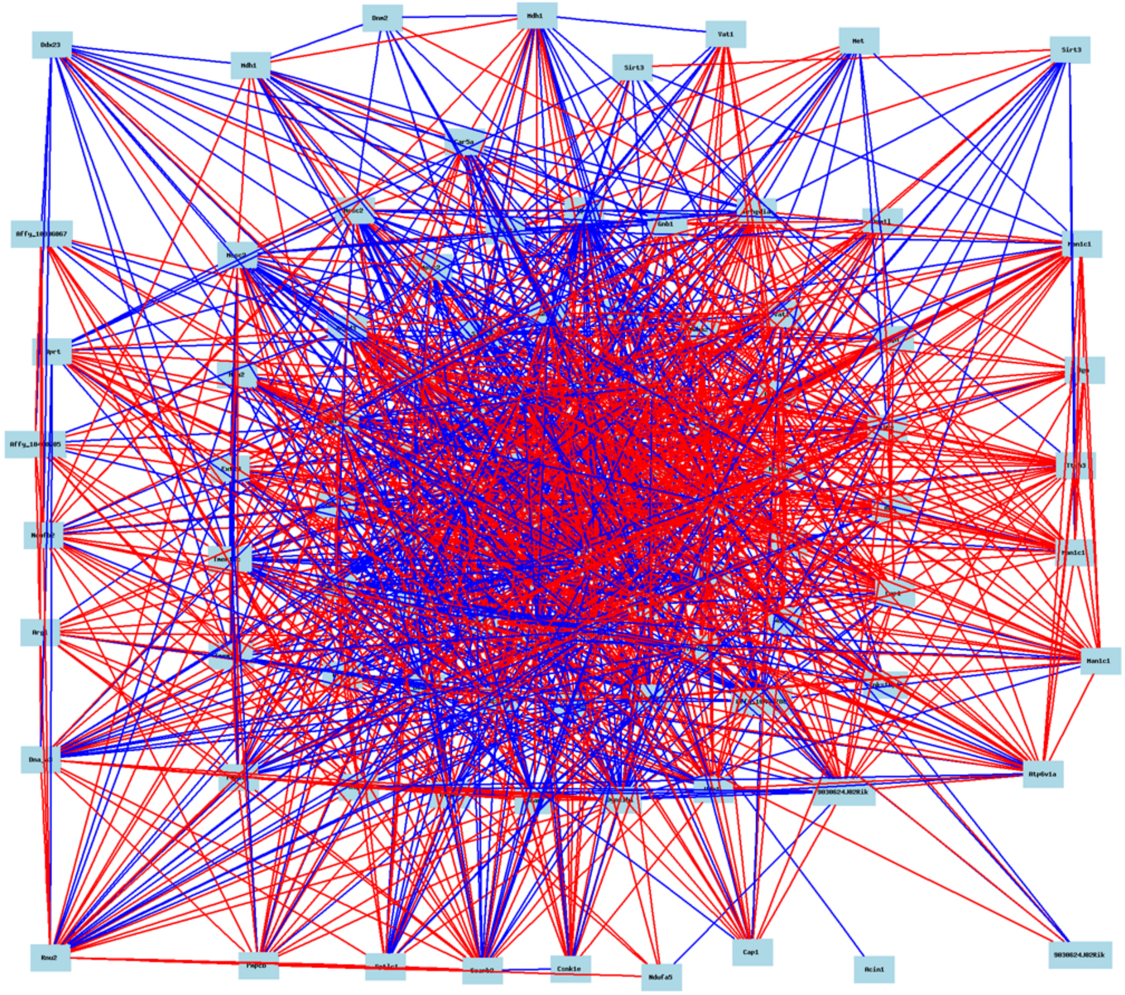

(B) Sirt3 PID# 10569006 co-expresion network

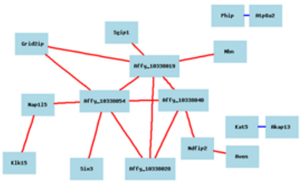

Figure S4. Network of 100 topmost correlated genes using Sirt3 exonic probe ID# 10568999 (**A**) and ID# 10569006 (**B**) in Liver SUH dataset (DS1). Red edges denote a Pearson correlation value > 0.8, whereas blue edges denote a Pearson correlation value < -0.8.

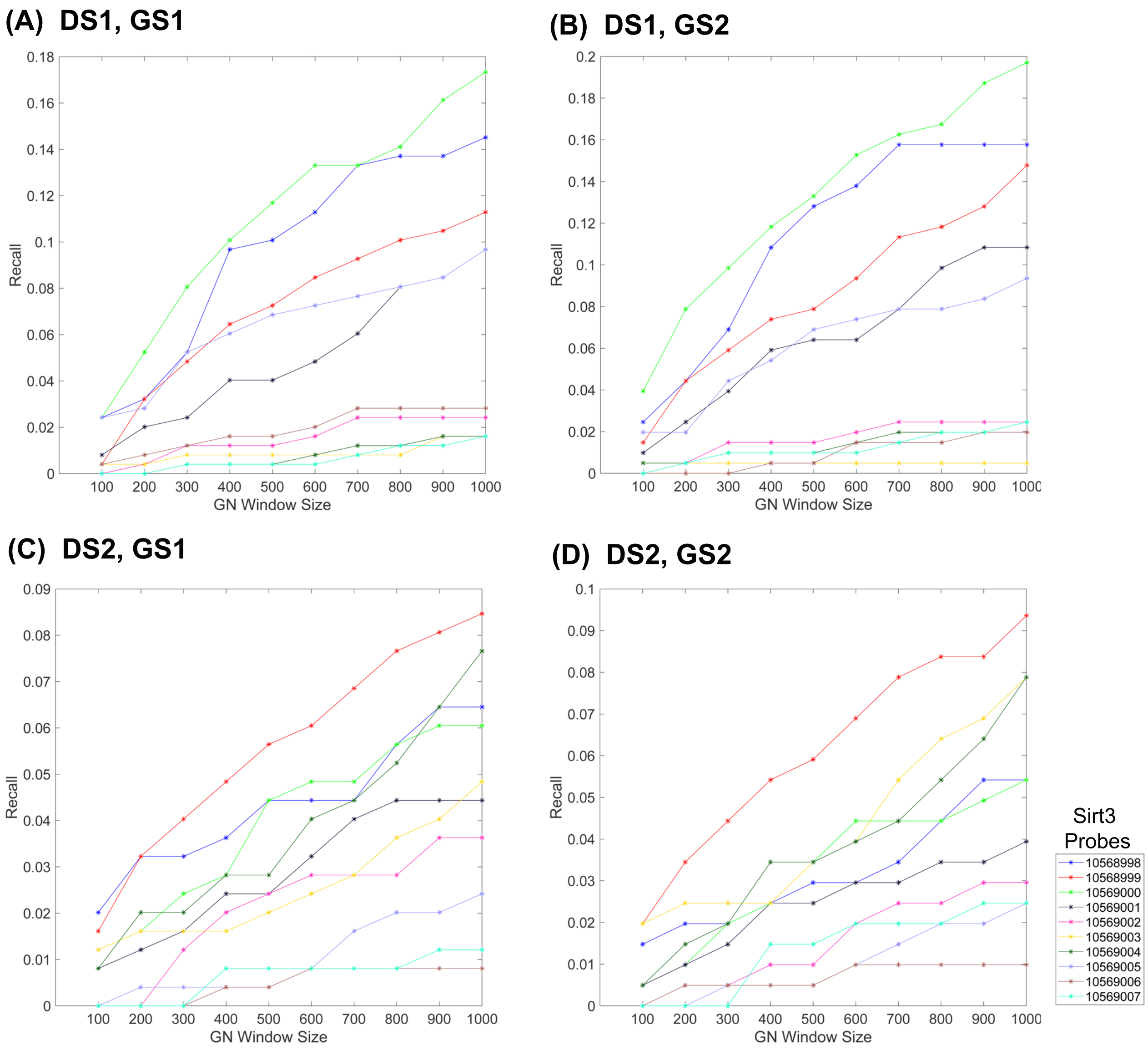

Figure S5. Relationship between gold standard recall and gene co-expression network window size. The recall values were calculated for the top 100-1000 co-expressed genes obtained from each Sirt3 probe in two different liver datasets (DS): 1) SUH BXD CCL4 Affymetrix Gene 1.0 ST treated (**A, B**) and 2) EFPL/LISP BXD CD+HFD Affymetrix Gene 1.0 ST (**C, D**). Recall values were calculated using two different Sirt3 gold standard gene sets: GS1, (**A, C**) and GS2, (**B, D**).

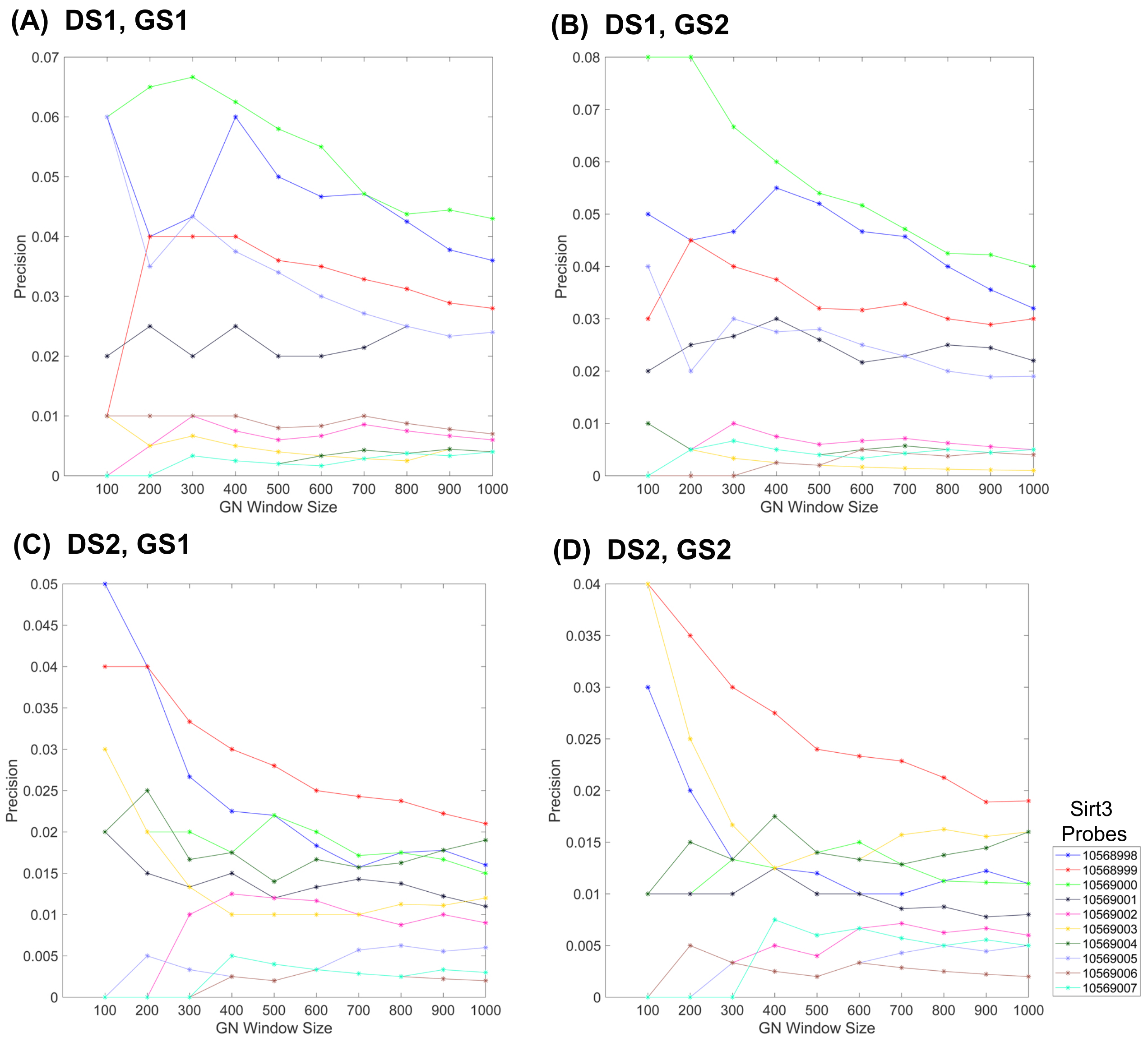

Figure S6. Relationship between gold standard precision and gene co-expression network window size. The precision values were calculated for the top 100-1000 co-expressed genes obtained from each Sirt3 probe in two different liver datasets (DS): 1) SUH BXD CCL4 Affymetrix Gene 1.0 ST treated (**A, B**) and 2) EFPL/LISP BXD CD+HFD Affymetrix Gene 1.0 ST (**C, D**). Precision values were calculated using two different Sirt3 gold standard gene sets: GS1, (**A, C**) and GS2, (**B, D**).

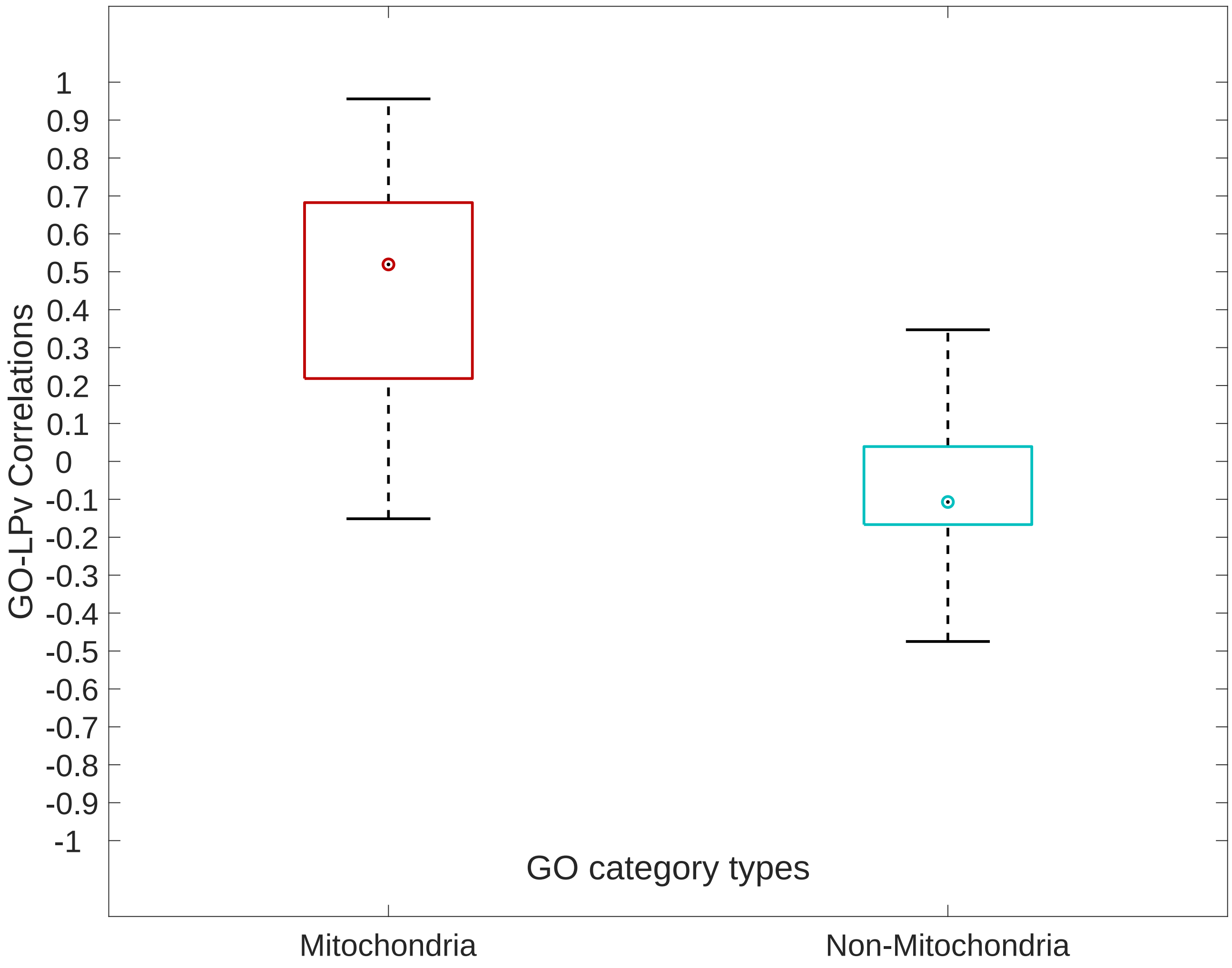

Figure S7. Box plots showing distribution of Pearson correlation coefficients between LPv and enrichment p-values for 2 different sets of GO categories across the 26 Sirt3 probe correlated top 500 genes. The GO category sets are: 71 categories containing either of terms 'mitochondria', 'mitochondrion', or 'mitochondrial' in their description (left); and 3061 categories that do not contain the aforementioned terms (right).

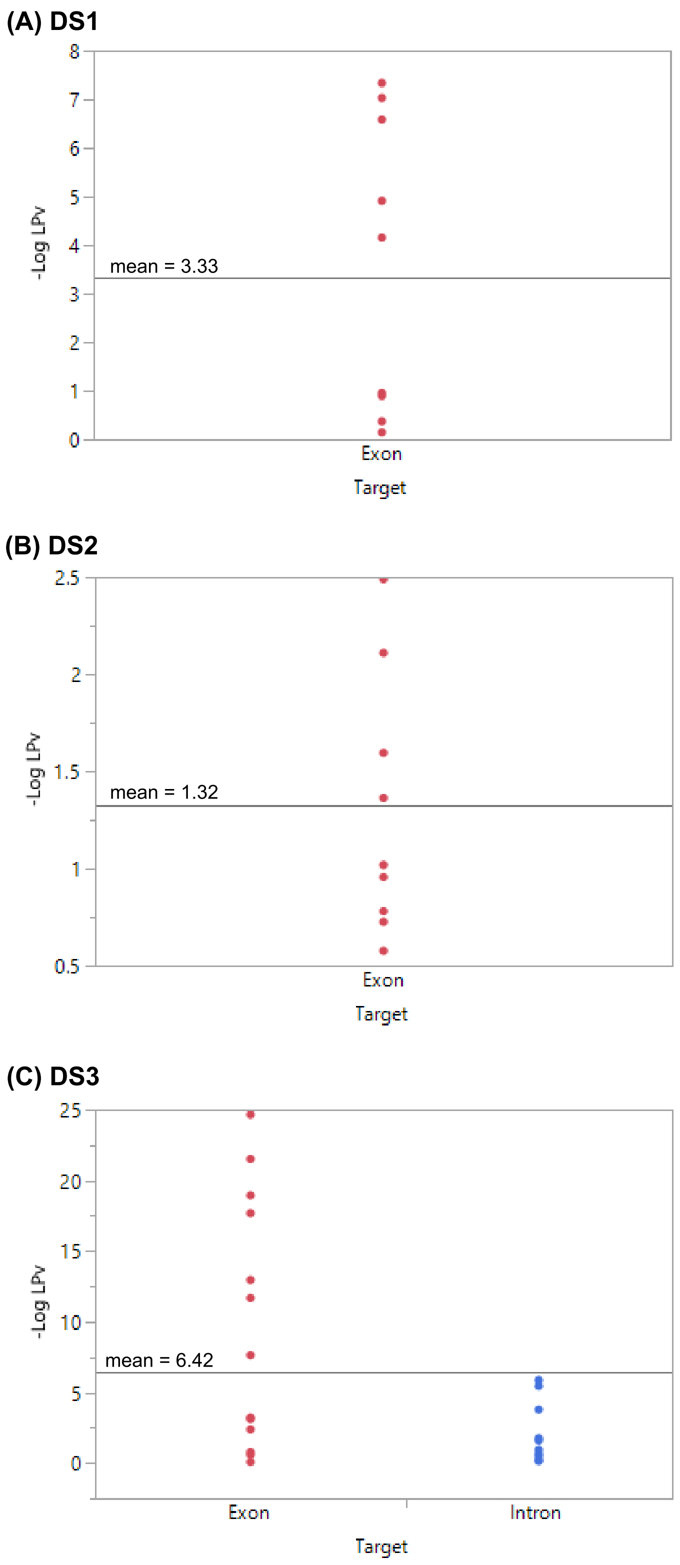

Figure S8. Line plots showing distribution of LPvs for Sirt3 probe correlated top 500 genes for datasets (A) DS1, (B) DS2 and (C) DS3.
